# Morphology, Anatomy and Secondary Metabolites Investigations of *Premna odorata* Blanco and Evaluation of Its Anti-Tuberculosis Activity Using In Vitro and In Silico Studies

**DOI:** 10.3390/plants10091953

**Published:** 2021-09-18

**Authors:** Fadia S. Youssef, Elisa Ovidi, Nawal M. Al Musayeib, Mohamed L. Ashour

**Affiliations:** 1 Department of Pharmacognosy, Faculty of Pharmacy, Ain-Shams University, Abbasia, Cairo 11566, Egypt; 2 Department for the Innovation in Biological, Agrofood and Forestal Systems, Tuscia University, 01100 Viterbo, Italy; eovidi@unitus.it; 3 Department of Pharmacognosy, College of Pharmacy, King Saud University, Riyadh 11495, Saudi Arabia; nalmusayeib@ksu.edu.sa

**Keywords:** anatomy, anti-tuberculous activity, Lamiaceae morphology, molecular docking, *Premna odorata*, secondary metabolites

## Abstract

In-depth botanical characterization was performed on *Premna odorata* Blanco (Lamiaceae) different organs for the first time. The leaves are opposite, hairy and green in color. Flowers possess fragrant aromatic odors and exist in inflorescences of 4–15 cm long corymbose cyme-type. In-depth morphological and anatomical characterization revealed the great resemblance to plants of the genus *Premna* and of the family Lamiaceae, such as the presence of glandular peltate trichomes and diacytic stomata. Additionally, most examined organs are characterized by non-glandular multicellular covering trichomes, acicular, and rhombic calcium oxalate crystals. *P. odorata* leaves *n-*hexane fraction revealed substantial anti-tuberculous potential versus *Mycobacterium tuberculosis*, showing a minimum inhibition concentration (MIC) of 100 μg/mL. Metabolic profiling of the *n-*hexane fraction using gas-chromatography coupled to mass spectrometry (GC/MS) analysis revealed 10 major compounds accounting for 93.01%, with *trans-*phytol constituting the major compound (24.06%). The virtual screening revealed that *trans*-phytol highly inhibited MTB C171Q receptor as *M. tuberculosis* KasA (*β-*ketoacyl synthases) with a high fitting score (∆G = −15.57 kcal/mol) approaching that of isoniazid and exceeding that of thiolactomycin, the co-crystallized ligand. Absorption, distribution, metabolism, excretion and toxicity predictions (ADME/TOPKAT) revealed that *trans-*phytol shows lower solubility and absorption levels when compared to thiolactomycin and isoniazid. Still, it is safer, causing no mutagenic or carcinogenic effects with higher lethal dose, which causes the death of 50% (LD50). Thus, it can be concluded that *P. odorata* can act as a source of lead entities to treat tuberculosis.

## 1. Introduction

*Premna* L. is a plant genus that was previously classified as a member of Verbenaceae [1], and recently, it has been moved to the family Lamiaceae and belongs to the subfamily Viticodeae [2]. *Premna* comprises about 200 species natively growing in Australia, Africa, subtropical and tropical Asia, and the Pacific Islands [2]. The term *Premna* is taken from the Greek word “premon”, which means tree stump, reflecting the twisted and short trunks of *P. serratifolia* L., the first discovered species of this genus. Members of this genus are characterized morphologically by being shrubs or trees, rarely pyroherbs as *P. herbacea* Roxb or lianas as *P. trichostoma* Miq. [3].

Certain *Premna* species are popular by having young twigs accompanied by small scales of triangular shapes and decussate arrangement present at the base and promptly fall when the branch becomes older. Most *Premna* species carry hairy leaves arranged in a decussate manner with the presence of a ridge among the petioles. Regarding the shape of the calyx, two types are present; one possesses four isomorphic lobes that are kept unchanged during the development of the flower and the formation of the fruits; meanwhile, the other is heteromorphic and possesses from zero to five lobes. In addition, the genus is characterized by two types of fruits, one is globose drupe-like fruit with fleshy mericarps, and in each mericarp there is one seed; however, the other is nearly clavoid with drupe-like single-seeded fruit with a fleshy mericarp [4].

Genus *Premna* is popular by the predominance of secondary metabolites belonging to various classes, including iridoid glycosides, diterpenoids, and phenylethanoids, lignans, sesquiterpenes, ceramides, megastigmanes and glyceroglycolipids. The richness in phytoconstituents is reflected in the biological activities of *Premna* species that show a wide array of biological effectiveness, represented mainly by their immunomodulatory, antimicrobial, anti-hyperglycemia, anti-inflammatory, cytotoxic activities [3,5].

*P. odorata* Blanco is represented mainly by small trees rarely reaching 10 m and was used in traditional medicine for vaginal irrigation and tuberculosis treatment. Flavonoids, iridoid glycosides and essential oils were isolated from *P. odorata* leaves and showed anti-aging and anti-tuberculosis activity [5,6].

Herein, the morphological and anatomical characters of fresh plant leaves, petiole, old and young stems, their histological sections, and air-dried finely powdered samples were comprehensively studied for the first time. Metabolic profiling of secondary metabolites in the *n*-hexane fraction obtained from the leaves was performed using GC/MS analysis. Furthermore, evaluation of the anti-tuberculous activity of *n*-hexane fraction using in vitro and in silico assays. In silico studies were performed on major metabolites identified from the *n*-hexane fraction on MTB C171Q receptor as KasA (*β*-ketoacyl synthases) to provide a solid support to consolidate what was previously reported in the literature about its anti-tuberculosis activity, which was also highlighted for the first time. Besides, ADME/TOPKAT predictions, major metabolites were identified from the *n*-hexane fraction were performed to highlight their pharmacodynamic, pharmacokinetic behavior and toxic potential.

## 2. Results and Discussion

### 2.1. Botanical Investigations

#### 2.1.1. Macromorphological Characterization

**Figure 1:**
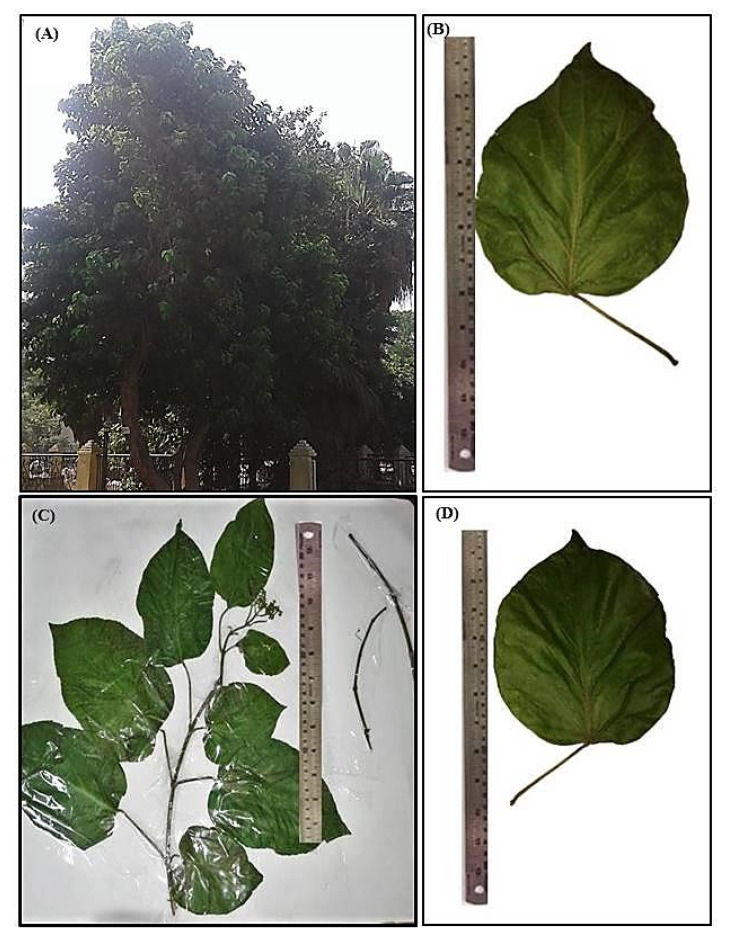
Morphological characterization of *P. odorata* displaying (**A**) entire tree, (**B**) leaf lower surface (×0.25), (**C**) leafy branch (×0.17) and (**D**) leaf upper surface (×0.25).

*P. odorata* Blanco (Lamiaceae) is an evergreen small tree or shrub nearly 10 m tall with diameter breast height (DBH) ranging between 15–30 cm. The leaves are opposite, hairy and green in color. Flowers exist in inflorescences of 4–15 cm long that are of corymbose cyme type. The flowers are pale green, yellowish or white with a fragrant aromatic odor. It flowers all year; meanwhile, fruit production occurs between March and November. It displays monopodial branching (Figure 1A).

##### Leaf

The leaves are oppositely arranged as simple, exstipulate, cauline, pubescent and petiolate. It is green in color, either old or young. It has variable shapes, either ovate, obovate, rotundate, to lanceolate, of 7–20 cm in length and 4–13.5 cm in width, with petioles of 20–80 mm long, velutinous to sparsely hairy. The leaf is characterized by an acuminate apex, emarginate to cordate base, serrate to entire margins. Both surfaces of the leaf are covered with hairs; meanwhile, venation of the leaf is tri-veined, arising from the base, with 3–7 main side veins. Its texture is subchartaceous or membranous and pubescent with a characteristic fragrant odor (Figure 1B–D).

##### Stem

Young stems are cylindrical, hairy and light brown, showing monopodial branching and opposite phyllotaxis; the old stems are erect, woody, and cylindrical with hairy texture and darker in color. Both young and old stems are brittle, break with a short fracture, and show a white solid interior and have nodes and internodes that are 7–9 cm in length. They have a fragrant odor and a characteristic taste (Figure 1C).

#### 2.1.2. Micromorphological Characterization

##### Leaf

Lamina

**Figure 2:**
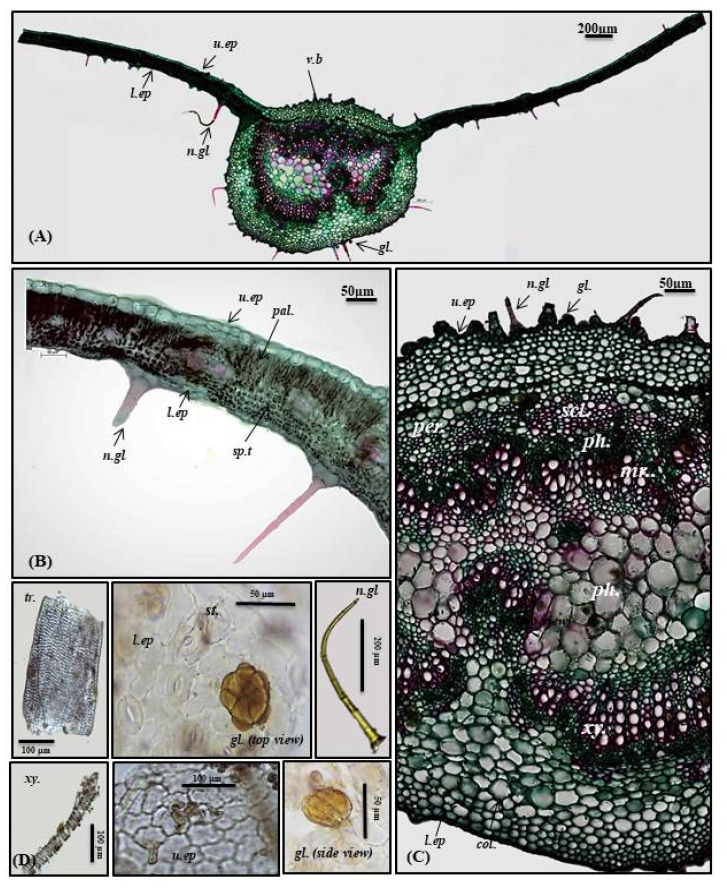
Micromorphology of *P. odorata* leaf showing (**A**) entire T.S (×100), (**B**) lamina (×400), (**C**) midrib region (×400), and (**D**) isolated elements. *Col.*, collenchyma; *gl.*, glandular peltate trichome; *l.ep.*, lower epidermis; *Mr.*, medullary rays; *n.gl.*, non-glandular trichome; *pal.*, palisade; *per.*, pericycle; *ph.*, phloem; *scl.*, sclerenchyma; *sp.t.*, spongy tissue; tr., tracheids; *u.ep*, upper epidermis; *v.b.*, vascular bundle; *xy.*, xylem.

The transverse cross-section in *P. odorata* leaf displayed the existence of upper and lower epidermis thickened with cuticle. The upper epidermis showed big, rectangular or cuboid-shaped cells; meanwhile, the lower epidermis displayed smaller cuboid-shaped cells than those of the upper epidermis. Stomata of the diacytic type are more prevalent in the lower epidermis. The lamina is characterized by the presence of dorsiventral mesophyll that is heterogeneous and discriminated into palisade and spongy layers. Below the upper epidermis, 1–2 layers of palisade cells that are highly compact cylindrical parenchymal cells and continuous in the midrib region in the form of a single layer of small cells. Palisade cells are characterized by the presence of narrow intercellular spaces and show green plastids. The spongy tissue comprises 4–5 layers of spherical parenchyma cells displaying intercellular spaces and interrupted by vascular bundles in small lateral branches. Two types of trichomes are present, with more prevalence on the lower epidermis, and appear more clearly in the powdered form. The first is non-glandular multicellular covering trichome with a 2-celled base. Meanwhile, the second is glandular peltate trichome (Figure 2A,B,D).

Midrib

It is convex in the upper surface of the leaf (adaxial side), possessing epidermis in a single layer, with cells cuboid in shape, thickened with cuticle. The upper epidermis is followed by 5–6 layers of collenchyma cells, and the cortical tissue is formed of several rows of collenchyma cells facing the lower epidermis with acicular and rhombic crystals of calcium oxalate. A large bowel-shaped collateral vascular bundle exists in the center of the midrib, crossed by medullary rays formed of elongated lines in the form of uni- to biseriate lines. The vascular bundles are formed of phloem and xylem. The former is composed of thin-walled cells of parenchyma; meanwhile, the latter is composed of xylem vessels, fibers with wide lumina, blunt apices, and wood parenchyma. The xylem is completely lignified and revealed a red color upon treatment with concentrated hydrochloric acid and phloroglucinol. The xylem vessels are thickened spirally, whereas wood parenchyma is composed of elongated cells with lignified pitted walls. The phloem area is surrounded by sclerenchyma patches, while the ground tissue is made of parenchyma occupying its center with abundant acicular and rhombic crystals of calcium oxalate. Arrangement of the xylem vessels radially occurs with the metaxylem directed toward the periphery (abaxial surface), while the protoxylem is directed toward the center (adaxial surface). Abundant crystals of calcium oxalate are also present. Numerous non-glandular multicellular covering trichomes and glandular peltate trichomes exist on the upper and lower epidermis (Figure 2A,C,D).

##### Petiole

**Figure 3:**
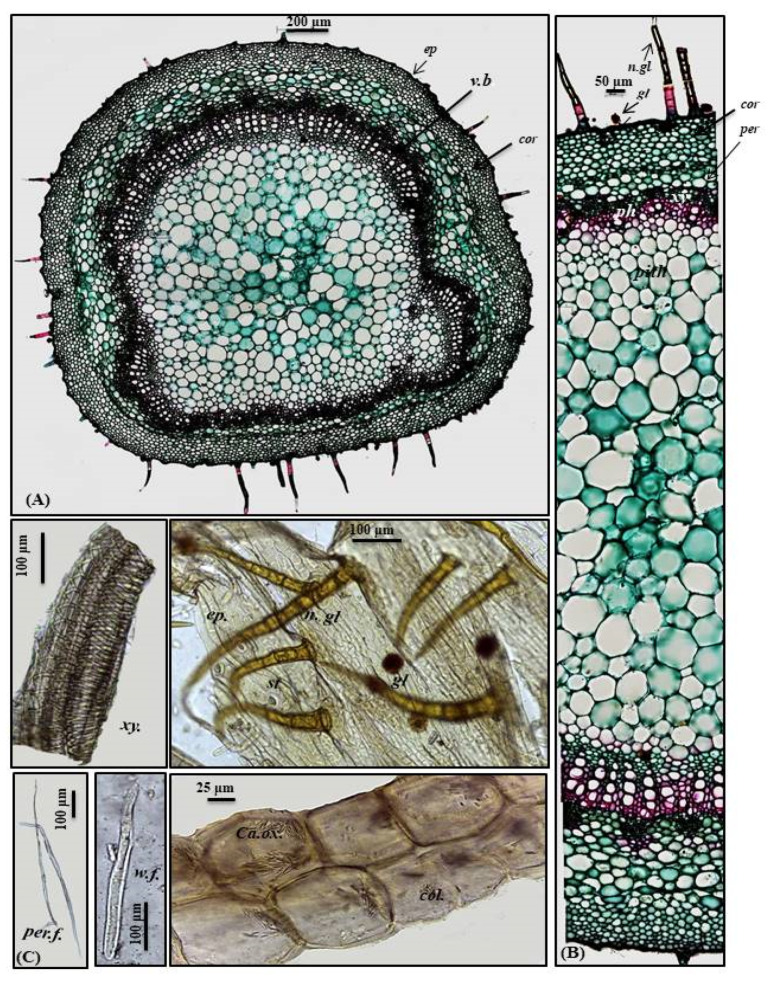
Micromorphology of *P. odorata* petiole showing (**A**) entire T.S (×100), (**B**) a part of T.S (×400) and (**C**) isolated elements. *Ca.ox.*, acicular crystals of calcium oxalate; *cor.*, cortex; *col.*, collenchyma; *ep.*, epidermis*; gl.*, glandular peltate trichome; *n.gl.*, non-glandular trichome; *per.*, pericycle; *per.f.*, pericycle fiber; *ph.*, phloem; *v.b*, vascular bundle; *w.f.*, wood fiber; *xy.*, xylem.

A transverse section obtained from the petiole revealed a cubical outline formed by a single layer of cubical thickened cells with diacytic stomata accompanied by both non-glandular and glandular peltate trichomes. Below the epidermis, the cortex comprises 6–7 layers of small-sized thick-walled angular collenchymatous cells followed by 4–5 layers of large-sized polygonal collenchyma cells containing abundant rhombic calcium oxalate crystals and showing sinuous cell walls. The petiole has collateral vascular bundles extending radially, taking a circular shape on the ventral side and a bowl shape on the dorsal side. They consist of an outer phloem and inner xylem, with the metaxylem directed toward the periphery (abaxial surface), with the protoxylem directed toward the center (adaxial surface). Vascular bundles are separated by medullary rays formed of elongated lines in the form of uni- to biseriate lines. As in the leaves, phloem is composed of thin-walled parenchyma cells; meanwhile, the xylem is composed of xylem vessels, fibers with wide lumina and blunt apices and wood parenchyma. The xylem is completely lignified and revealed a red color upon treatment with concentrated hydrochloric acid and phloroglucinol. The xylem vessels are thickened spirally, whereas wood parenchyma is composed of elongated cells with lignified pitted walls. The ground tissue existing in the central region of the petiole is composed of big parenchyma cells with few scattered rhombic calcium oxalate crystals (Figure 3A–C).

##### Stem

Young stem

**Figure 4:**
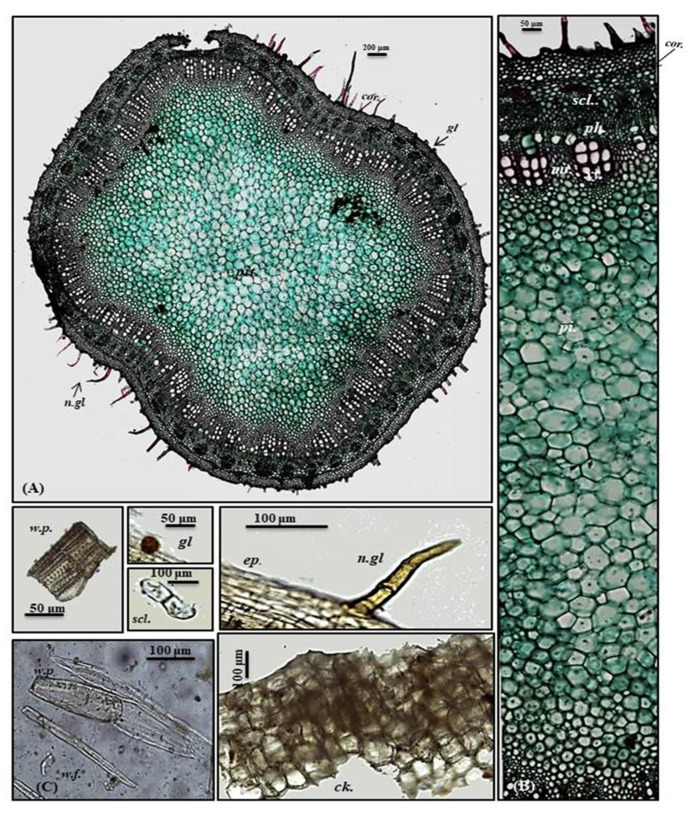
Micromorphology of *P. odorata* young stem branch showing (**A**) entire T.S (×100), (**B**) a part of T.S (×400) and (**C**) isolated elements. *Ck.*, cork (old stem branch); *cor.*, cortex; *ep.*, epidermis*; gl.*, glandular peltate trichome*; n*.*gl.*, non-glandular trichome*; per.*, pericycle; *per.f.*, pericycle fiber; *pi.*, pith; *ph.*, phloem; *scl.*, sclerenchyma; *tr.*, tracheid; *w.f.*, wood fiber; *w.p.*, wood parenchyma.; *xy.*, xylem.

A transverse section obtained from the young stem revealed a quasi-square outline with a slight curvature in the middle of only two opposite sides composed of epidermis, narrow cortex and vascular bundles. It is covered by both non-glandular and glandular peltate trichomes. The epidermal cells are present in only a single layer, and they are nearly square in appearance that is densely arranged and thickened with cuticle having diacytic stomata. The cortex is narrowly composed of 5–6 rows of nearly circular thickened collenchyma cells ending in a pericycle surrounding the vascular bundles. The pericycle is continuously formed of 1–2 rows of polygonal small, highly thickened, densely arranged cells. The vascular region is continuously composed of open collateral vascular bundles with phloem, cambium, and xylem traversed by medullary rays. Additionally, the phloem area is surrounded by sclerenchyma patches, and the xylem is lignified formed of spirally thickened xylem vessels. The pith represents a big part of the total stem diameter consisting of large, polygonal, thin-walled cells containing acicular crystals of calcium oxalate (Figure 4A,B).

Old stem

**Figure 5:**
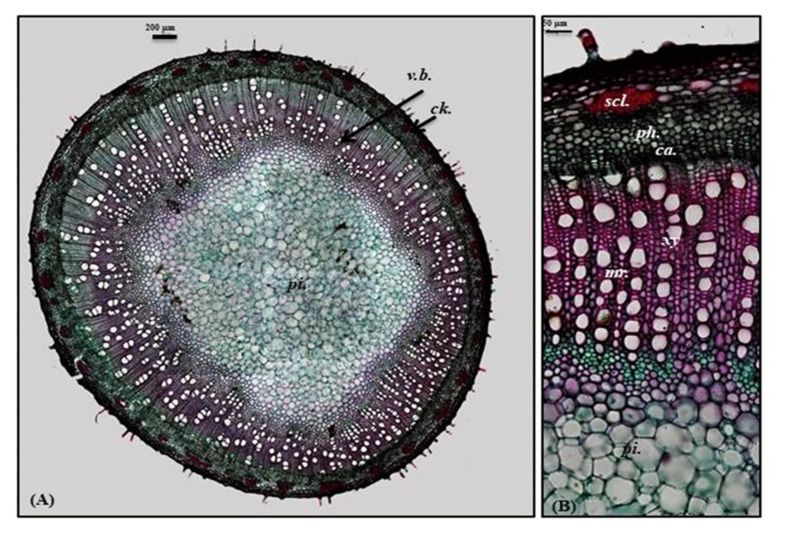
Micromorphology of *P. odorata* old stem branch showing (**A**) entire T.S (×100) and (**B**) a part of T.S (×400) *Ca.*, cambium; *Ck.*, cork; *scl.*, sclerenchyma*; pi.*, pith; *ph.*, phloem; *xy.*, xylem.

A transverse section obtained from the old stem revealed a nearly circular outline covered with non-glandular and glandular peltate trichomes. It is composed of cork cells followed by cork cambium and a narrow secondary cortex composed of patches of sclerenchyma, particularly above the phloem in a well-developed vascular system. The cork is formed of elongated brown cells, lignified and thickened cells, which is absent in the young stem. The vascular region is continuously composed of open collateral vascular bundles with phloem, cambium, and xylem traversed by medullary rays. The xylem occupies a wider area than in the young stem, followed by pith that is relatively narrower with respect to the young stem. It comprises nearly circular thin-walled large parenchymatous cells (Figure 5A–C). The microscopical measurements of the various elements existing in the leaves, petiole and stems of *P. odorata* are illustrated in Table 1. In-depth, comprehensive botanical study of *P. odorata* leaf, petiole and stems revealed its great resemblance to other members in genus *P. odorata* and Lamiaceae Family, such as the presence of glandular trichomes and diacytic stomata. The presence of diacytic stomata, covering trichomes that are unicellular or uniseriate, simple or branched as well as the glandular trichomes such as peltate or capitate hairs with unicellular stalk and unicellular or multicellular head usually formed of 8 cells radiating from the stalk that are abundant on all vegetative parts, are among the common anatomical features of all Lamiaceae species such as *Mentha piperita* L., *Lavandula officinalis* L., *Ocimum basilicum* L. and *Thymus vulgaris* L. as well [7,8]. Although few studies have been conducted to explore the anatomy of many *Premna* species, investigations performed on *P. serratifolia* showed great similarity with *P. odorata* in virtue of possessing sclerenchyma cells in the form of patches above the phloem, the vascular bundle in the region of the midrib takes the shape of the bowel and the stomata is diacytic in addition to glandular peltate trichomes [9].

### 2.2. Metabolic Profiling of the Leaves n-Hexane Fraction Using GC/MS Analysis

**Figure 6:**
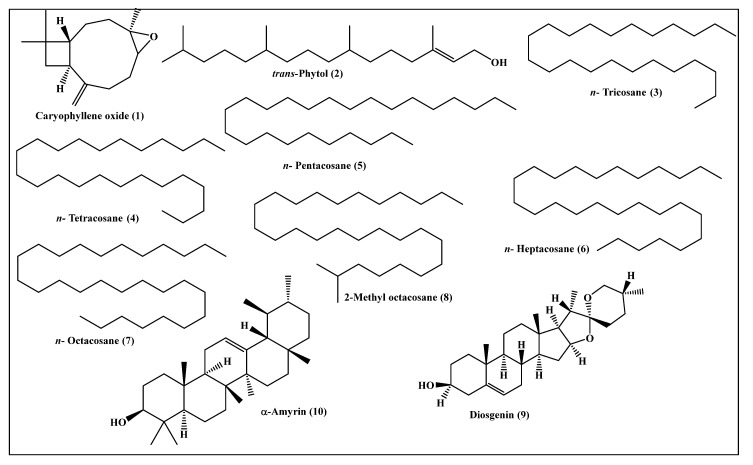
Scheme showing the compounds identified from the *n*-hexane fraction of *P. odorata* leaves using GC/MS analysis.

Metabolic profiling of the *n*-hexane fraction obtained from the leaves of *P. odorata* leaves using GC/MS analysis revealed the presence of 10 major compounds accounting for 93.01%. They belong mainly to oxygenated sesquiterpenes, higher alkanes and steroidal compounds. *trans*-Phytol constitutes the major compound (24.06%), followed by *n*-octacosane (15.28%) and α-amyrin (13.37%). Compounds identified from the *n*-hexane fraction of *P. odorata* leaves using GC/MS analysis are illustrated in Figure 6 and Table 2.

**Table 2:** Compounds identified from the *n*-hexane fraction of *P. odorata* leaves using GC/MS analysis.

**Heading**	**Compound**	**RI**		**Content [%]**	**Identification Method**
		***Exp.***	***Pub.***		
1	Caryophyllene oxide	1576	1576 [10]	7.96	MS, RI
2	*trans*-Phytol	2096	2101 [11]	24.06	MS, RI
3	*n*-Tricosane	2280	2300 [12]	3.15	MS, RI
4	*n*-Tetracosane	2404	2400 [12]	3.20	MS, RI
5	*n*-Pentacosane	2455	2500 [12]	9.69	MS, RI
6	*n*-Heptacosane	2669	2700 [12]	4.49	MS, RI
7	*n*-Octacosane	2798	2800 [12]	15.28	MS, RI
8	2-Methyl octacosane	2854	2857 [13]	2.53	MS, RI
9	Diosgenin	3276	3220 [14]	9.28	MS, RI
10	α-Amyrin	3384	3382 [15]	13.37	MS, RI
	**Total identified**			**93.01**	

### 2.3. Evaluation of the Anti-Tuberculous Activity of the Leaves n-Hexane Fraction

*P. odorata* leaves *n-*hexane fraction revealed substantial anti-tuberculous potential versus *M. tuberculosis*, showing a minimum inhibition concentration (MIC) of 100 μg/mL, whereas that of isoniazid showed MIC of 25 μg/mL. Furthermore, *P. odorata* leaves *n-*hexane fraction and isoniazid inhibited *M. tuberculosis* growth by 71% and 93% at 12.5 μg/mL, respectively. This activity is following research that previously reported the anti-tuberculous effect of *P. odorata.* A study conducted on the leaves crude methanol extract revealed poor inhibition versus *M. tuberculosis*, with MIC value of more than 128 μg/mL; meanwhile, the inhibitory potency increased in different fractions, particularly dichloromethane fraction and its subfractions, to reach MIC values ranging between 54 to 120 µg/mL [16]. Additionally, MMA-ELISA (*Mycobacterium tuberculosis* antigen ELISA technique) revealed that the volatile oils isolated from leaves, flowers and young stems of *P. odorata* exhibited anti-TB activities in a dose estimated by 100 µL/mL in vitro and 300 µL/mL in vivo when tested separately. This activity increased significantly upon using a combination of them [17]. This was further consolidated by an additional study that showed that the TLR-4/NFκB signaling pathway is involved in the immunomodulatory effects triggered by the volatile oils isolated from *P. odorata* different organs versus TB infection in addition to their antioxidant effects [18]. Isoniazid is a pro-drug that exerts its anti-tuberculous activity after being activated in isoniazid-susceptible mycobacterial species. It significantly prohibits the formation of cell wall mycolic acids that constitutes the main component forming the envelope of *M. tuberculosis*. This may be attributed to its effect on certain enzymes that are involved in the synthesis of mycolic acids such as the fatty-acid enoyl-acyl carrier protein reductase (InhA), a complex of an acyl carrier protein (AcpM) and a β-ketoacyl-ACP synthase (KasA). Mutations have been found in the promoter regions, or less commonly, in the genes that encode these proteins in *M. tuberculosis*. These proteins decrease in strains exhibiting low resistance to isoniazid and increase in strains showing resistance to *M. tuberculosis*, suggesting that this could be an isoniazid mode of action [19].

### 2.4. In Silico Molecular Modeling Study

**Figure 7:**
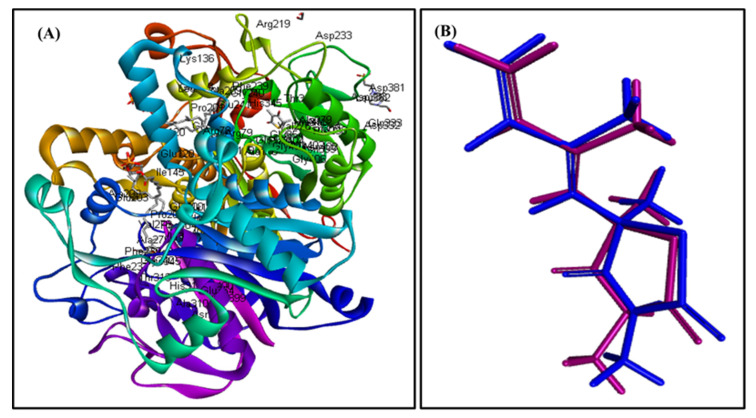
MTB C171Q receptor KasA inhibitor ribbon structure (**A**); validation of the docking experiment (**B**).

**Figure 8:**
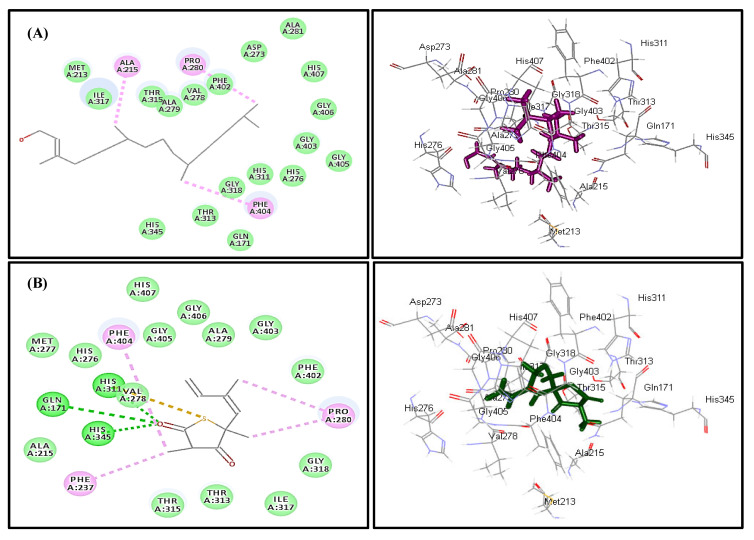
Two- and three-dimensional binding behavior of *trans*-phytol (**A**) and thiolactomycin; the co-crystalized ligand (**B**) within MTB C171Q receptor KasA inhibitor (4C6X) active site using C-docker protocol.

In silico molecular docking inhibition study of the identified compounds was performed on MTB C171Q receptor as KasA (*β-*ketoacyl synthases) inhibitor (PDB ID 4C6X; 1.95 Å) that represents one of the attractive therapeutic targets to combat *M. tuberculosis* (Table 3). Root mean square deviation (RMSD) value between the co-crystallized ligand (thiolactomycin) docked within the pocket of the active center and the original molecule co-crystallized with the molecule equals 0.47, indicating the validity of the docking process (Figure 7). Virtual screening studies revealed that *trans*-phytol (**2**) highly inhibited the protein with a high fitting score (∆G = −15.57 kcal/mole) approaching that of isoniazid (∆G = −21.47 kcal/mole) and exceeding that of thiolactomycin, the co-crystallized ligand (∆G = −13.03 kcal/mole). This firm fitting is due to the size of the molecule in addition to the formation of three π-alkyl bonds with Phe404, Pro280, Ala215 and Van der Waals interaction with many amino acid residues existing at the active site (Figure 8).

Many studies previously conducted on *trans*-phytol revealed its significant effect as an anti-tuberculous drug [20]. *trans*-Phytol isolated as a principal component from *Leucas volkensii* Gürke (Labiatae) displayed MIC value equals to 2 µg/mL versus *M. tuberculosis* approaching that of ethambutol, a clinically useful drug showing MIC value between 0.95 and 3.8 µg/mL [21].

### 2.5. ADME/TOPKAT Prediction

**Figure 9:**
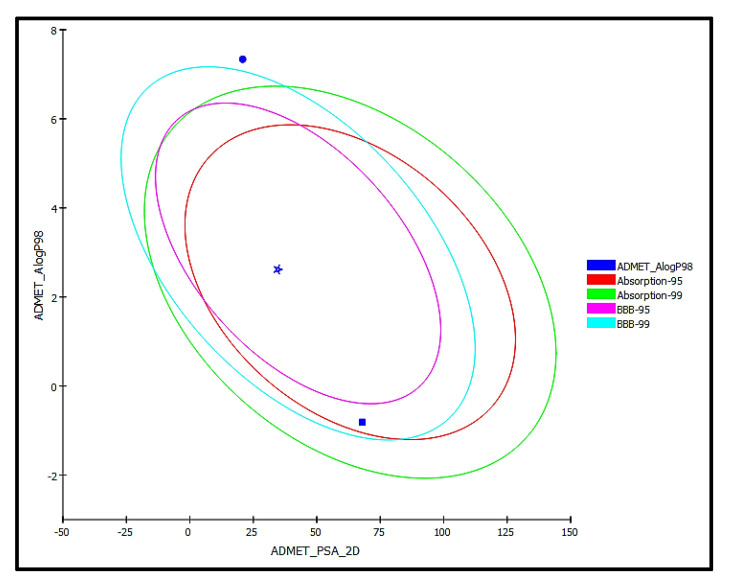
ADMET Plot for bioactive compound identified in *n*-hexane fraction of *P. odorata* leaves showing the 95% and 99% confidence limit ellipses corresponding to the blood-brain barrier (BBB) and the human intestinal absorption models; *trans*-phytol (filled circle); Co-crystalized ligand (Thiolactomycin) (star); and isoniazid (filled square) in ADMET_AlogP98.

The pharmacodynamic and pharmacokinetic behavior of *trans*-phytol, the most bioactive compound identified from the *n*-hexane fraction of *P. odorata* leaves as revealed by docking experiments, was determined. This was performed via ADME/TOPKAT prediction using Discovery Studio 4.5 (Accelrys Inc., San Diego, CA, USA) and was compared with the properties of thiolactomycin (co-crystallized ligand) and isoniazid (a standard anti-tuberculous drug) (Table 4). *trans*-Phytol is possibly soluble with low human intestinal absorption and unknown blood brain barrier (BBB) penetration level. Thus, it lies outside the 99% absorption ellipse, as shown in the ADMET plot (Figure 9). It shows more than 90% plasma protein binding (PBB), showing no hepatotoxicity and causes no inhibition to cytochrome P450 2D6 in contrast to isoniazid, which causes hepatotoxicity that was previously reported [22]. Additionally, *trans*-phytol is relatively safe being non-mutagen in chemical Ames mutagenicity test, non-carcinogen to male and female NPT (National Toxicology Program) rats with rat oral LD50 equals to 9.43 g/kg·bw in contrast to isoniazid that causes mutagenicity and is carcinogen to female rats. Additionally, it does not show ocular irritancy but moderate skin irritancy. Thus, it is obvious that although *trans*-phytol shows lower solubility and absorption levels compared to thiolactomycin and isoniazid, it is safer, causing no mutagenic or carcinogenic effects with higher LD50 with potent therapeutic effect. Therefore, it can be subjected to certain treatments to improve its pharmacodynamic and pharmacokinetic behavior to be incorporated in pharmaceutical dosage forms to treat tuberculosis. Regarding the carcinogenicity and mutagenicity of isoniazid, this comes in accordance with what is previously published, where isoniazid and procarbazine, two hydrazine derivatives, were evaluated for their DNA damage that can occur in male rabbits treated with these drugs. Procarbazine displayed high carcinogenicity and mutagenicity, whereas isoniazid revealed certain carcinogenic and mutagenic effects that appear in mice. This was further confirmed in mammalian test systems [23,24].

## 3. Materials and Methods

### 3.1. Plant Material

Stems and leaves of *P. odorata* Blanco (Lamiaceae) were provided from the zoo botanical garden, Giza, Egypt in May 2018. The geographical coordinates of the collection site are 30°1′28.32″ N; 31°12′50.03″ E, at 23 m altitude. Authentication was performed morphologically by taxonomy specialist Engineer Therease Labib, Consultant of Plant Taxonomy at the Ministry of Agriculture and Director of Orman Botanical Garden, Giza, Egypt. This was performed via comparing the morphological characters that can be detected by the naked eye or via using lens with low magnification power with the descriptions of the botanical drug listed in monographs or floras. These characters commonly used in morphological authentication include shape, color, and size of the leaf or fragments of it as well as fruits and flowers. Voucher specimens from *P. odorata* leaves and stems are maintained at the Pharmacognosy Department, Faculty of Pharmacy, Ain Shams University (PHG-P-PO-501).

### 3.2. Morphological and Anatomical Investigations of the Leaves, Petioles and Stems

Morphological studies were performed on air-dried specimens, employing the standard herbarium techniques. A canon camera (Canon PowerShot A580) was used to capture photographs of the entire plant organs. For anatomical investigations, the fresh plant samples were fixed in 70% ethanol containing 5% glycerin directly after being collected. A manual microtome (American Optical Company, model 900) was used to perform the histological sections, which were subsequently stained with malachite green and safranin. The cross-sections of the leaves, petioles, young and old stems, and the dried powdered samples were used to perform the botanical studies. Leica ICC50 HD camera integrated microscope (Carl Zeiss, Jena, German) was used to capture the photographs [25,26].

### 3.3. Preparation of the Leaves n-Hexane Fraction

In total, 100 g of air-dried *P. odorata* Blanco (Lamiaceae) leaves were ground into a fine powder and exposed to exhaustive maceration in neat methanol (3 × 1 L) and subsequently filtrated at 45 °C in vacuo using Rotavap (Buchi Rotavapor R-114 coupled with Buchi Vac V-500 pump, Switzerland) until completely dried and then lyophilized to give 9.8 g of total methanol extract. An amount of 9 g of the lyophilized powder was solubilized in 70% methanol and subsequently fractionated using 3 L of distilled *n*-hexane to give 0.5 g of *n*-hexane fraction [27].

### 3.4. Metabolic Profiling of the Leaves n-Hexane Fraction Using GC/MS Analysis

Gas chromatography coupled with mass spectrometry (GC/MS) analyses were performed on Shimadzu GCMS-QP 2010 (Shimadzu Corporation, Koyoto, Japan) provided by Rtx-5MS (30 m × 0.25 mm i.d. × 0.25 µm film thickness) capillary column (Restek, PA, USA) and attached to a Shimadzu mass spectrometer. The column temperature was initially set at 50 °C for 3 min. Then, the temperature was gradually increased from 50 to 300 °C at a rate of 5 °C/min and then isothermally maintained at 300 °C for 10 min. The temperature of the injector was kept at 280 °C. Helium was used as a carrier gas at a flow rate of 1.37 mL/min. Temperatures of 280 and 220 °C were the ion source and the interface, respectively. Injection of 1 µL of 1% *v/v* of diluted sample was achieved via a split mode adopting a split ratio of 15:1. Recording the mass spectrum was performed in EI mode of 70 eV from *m/z* 35 to 500. Compound quantitation was performed based on the normalization method, employing the reading of three chromatographic runs. Meanwhile, compound identification was made based upon the retention indices of the identified compounds with respect to a homologous series of *n*-alkanes (C8–C28) that are injected using the same conditions and by comparing mass spectra of the identified compounds with those reported in the National Institute of Standards and Technology (NIST) and Wiley library database in addition to the literature [28,29,30].

### 3.5. Evaluation of the Anti-Tuberculous Activity of the Leaves n-Hexane Fraction

The anti-tuberculous activity of *P. odorata* leaves *n-*hexane fraction was determined in vitro by microplate Alamar blue assay (MABA) using *M. tuberculosis* strain (RCMB 010126) obtained from the Regional Center for Mycology and Biotechnology, Cairo, Egypt, and adopting the method described by Gamal El-Din et al. (2018) Isoniazid was obtained from (Sigma Aldrich) as a standard anti-tuberculous drug. All the measurements were performed in triplicate at three independent times, and data were expressed as the mean ± SD [31,32].

The inhibition percent was calculated from the following equation:

1—(mean of test well/mean of B wells) ×100,

MIC: the minimum concentration of drug that prohibited color change.

### 3.6. In Silico Molecular Modeling Study

In silico molecular docking study of the identified compounds was performed on one of MTB C171Q receptors such as KasA inhibitor (PDB ID 4C6X; 1.95 Å), a crucial enzyme for mycobacterial survival downloaded from the protein data bank. This was performed employing Discovery Studio 4.5 software (Accelrys Inc., San Diego, CA, USA) by adopting C-Docker protocol as previously described [33,34,35]. To validate the docking process, the co-crystallized ligand (thiolactomycin) was docked within the pocket of the active center and compared with the original molecule co-crystallized with the molecule. The value of RMSD (root mean square deviation) indicated the validity of the process [32].

### 3.7. ADME/TOPKAT Prediction

ADME/TOPKAT (absorption, distribution, metabolism, excretion and toxicity) prediction was performed on the bioactive compound identified from the *n*-hexane fraction of *P. odorata* leaves, *trans*-phytol, in addition to thiolactomycin (co-crystallized ligand) and isoniazid. This was performed using Discovery Studio 4.5 (Accelrys Inc., San Diego, CA, USA). Human intestinal absorption, aqueous solubility, plasma protein binding prediction (PPB), blood-brain barrier penetration (BBB), hepatotoxicity level and cytochrome P450 (2D6) were chosen as ADME descriptors. Furthermore, rat oral LD50, carcinogenic affect female and male rat NPT (National Toxicology Program), skin and ocular irritation and Ames mutagenicity were chosen as toxicity parameters [36].

## 4. Conclusions

Botanical study of *P. odorata* leaf, petiole and stem showed its great resemblance to other members in *Premna* genus and Lamiaceae family, such as the presence of glandular peltate trichomes and diacytic stomata. *P. odorata* leaves *n-*hexane fraction revealed substantial anti-tuberculous potential versus *M. tuberculosis*. *trans-*Phytol constitutes the major compound in the *n-*hexane fraction of the leaves, as demonstrated by GC/MS analysis. *trans-*Phytol highly inhibited the MTB C171Q receptor as KasA (*β-*ketoacyl synthases), approaching isoniazid and exceeding that of thiolactomycin, the co-crystallized ligand, as revealed from the docking studies. ADME/TOPKAT predictions showed that *trans*-phytol possesses less solubility and absorption levels when compared to thiolactomycin and isoniazid, tested as standard molecules. Still, it is safer, causing no mutagenic or carcinogenic effects with higher LD50. Thus, *P. odorata* can act as a source of lead entities to treat tuberculosis. However, further studies are recommended to be performed on other fractions of the plant, such as the polar fractions and their subsequent isolated compounds to evaluate their biological activities. In addition, it is highly recommended to directly use *n*-hexane for the extraction, and thus more non-polar compounds will be extracted and concomitantly will increase the robustness of the study.

## Figures and Tables

**Table 1:** The microscopical measurements of the various elements existing in the leaves, petiole and stems of *P. odorata* (in µm).

Item	Length	Width	Height	Diameter
**Leaf**				
Upper epidermis	71.50–66.8–62.20	8.80–10.20–11.00	6.40–10.70–15.00	
Lower epidermis	30.45–40.18–49.90	45.60–10.10–14.60	5.20–5.50–6.80	
Palisade cells	20.30–25.47–30.64	5.60–6.80–7.20	15.40–16.67–17.80	
Stomata	21.45–25.50–29.55	13.90–16.50–19.10		
Non-glandular trichome	250.56–400.34–550.23	47.60–50.79–53.98		
Glandular (Peltate trichome)	39.60–42.86–46.12	30.59–35.70–40.81		
Xylem vessels				19.89–22.11–24.33
**Petiole**				
Epidermis	25.00–37.50–50.02	6.50–10.40–14.32	4.90–5.50–6.10	
Stomata	20.45–25.50–30.55	14.90–16.50–18.10		
Non-glandular trichome	300.45–375.52–450.60	46.60–51.79–56.99		
Glandular (Peltate trichome)	48.60–50.86–53.12	23.59–25.70–27.81		
Xylem vessels				28.89–34.61–40.33
Wood fibers	350.50–375.60–400.70	22.59–25.30–28.00		
Pericyclic fibers	640.44–684.70–708.95	7.73–10.42 -13.11		
**Young and old stem branch**				
Epidermis	25.76 –30.58–35.4	8.14–10.70–13.26	4.00–4.50–5.00	
Cork cells	48.68–49.61–50.54	48.68–49.61–50.54		
Non-glandular trichome	157.50–167.52–177.54	18.40–21.69–24.98		
Glandular (Peltate trichome)	47.60–49.86–52.12	22.39–25.30–28.21		
Xylem vessels				27.89–35.61–41.33
Wood fibers	320.50–375.40–430.30	23.59–26.30–29.00		
Wood parenchyma	200.00–210.00–220.00	22.00–25.50–29.00		

**Table 3:** Free binding energies (kcal/mole) of the identified compounds in the active site of MTB C171Q receptor KasA inhibitor using in silico studies.

Compound	MTB C171Q Receptor KasA Inhibitor (4C6X)	Number of Formed Hydrogen and π-Bonds with the Amino Acid Residues
Caryophyllene oxide (**1**)	8.04	5; Phe404, Thr313, Ile317, Ala279, Val278
*trans*-Phytol (**2**)	−15.57	3; Phe404, Pro280, Ala215
*n*-Tricosane (**3**)	FD	-
*n*-Tetracosane (**4**)	FD	-
*n*-Pentacosane (**5**)	FD	-
*n*-Heptacosane (**6**)	FD	-
*n*-Octacosane (**7**)	FD	-
2-Methyl octacosane (**8**)	FD	-
Diosgenin (**9**)	173.96	6; Phe237, Met213, Pro280, Ala215, Phe402, His311
α-Amyrin (**10**)	FD	-
Isoniazid	−21.47	4; Asp319, Gln322, His311, Pro280
Co-crystalized ligand (Thiolactomycin)	−13.03	6; Gln171, His345, His311, Val278, Phe404, Pro208

FD: Fail to dock, “-”. Means no hydrogen or π-Bonds are formed with the Amino Acid Residues; Positive values indicate unfavorable interaction.

**Table 4:** The absorption, distribution, metabolism, excretion, and toxicity (ADME/TOPKAT) predictions for bioactive compound identified from the *n*-hexane fraction of *Premna odorata* leaves, *trans*-phytol, thiolactomycin (co-crystallized ligand) and isoniazid.

Compounds	*trans*-Phytol	Thiolactomycin	Isoniazid
ADMET parameters			
Absorption Level	3	0	0
Solubility Level	2	3	4
BBB Level	4	3	1
PPB Level	True	True	False
CPY2D6	NI	NI	NI
Hepatotoxic	Non-toxic	Non-toxic	Toxic
PSA-2D	20.82	34.60	67.91
Alog p98	7.3	2.62	−0.81
TOPKAT parameters			
Ames prediction	Non-mutagen	Non-mutagen	Mutagen
Rat oral LD50 (g/kg·bw)	9.43	0.20	0.48
Rat female FDA	Non-carcinogen	Non-carcinogen	Carcinogen
Rat Male FDA	Non-carcinogen	Carcinogen	Non-carcinogen
Skin irritancy	Moderate	Moderate	None
Ocular irritancy	None	Mild	Mild

0, 1, 2, and 3 indicate good, moderate, low and very low absorption, respectively; 0, 1, 2, 3, 4, and 5 indicate extremely low, very low but possible, low, good, optimal, and too soluble, respectively; 0, 1, 2, 3, and 4 denote very high, high, medium, low, and undefined, penetration via BBB, respectively. PBB, plasma protein binding; FALSE means less than 90%, TRUE means more than 90%; NI, non-inhibitor.

## Data Availability

Data are available upon request from the first author.
